# Cytomorphometric Characteristics of Buccal Mucosal Cells in Behçet's Disease Patients

**DOI:** 10.1155/2016/6035801

**Published:** 2016-03-21

**Authors:** Erol Aktunc, Zehra Safi Oz, Sibel Bektas, Cevdet Altinyazar, Rafet Koca, Serdar Bostan

**Affiliations:** ^1^School of Medicine, Department of Family Medicine, Bulent Ecevit University, 67600 Zonguldak, Turkey; ^2^School of Medicine, Department of Medical Biology, Bulent Ecevit University, 67600 Zonguldak, Turkey; ^3^Department of Pathology, Taksim Education and Research Hospital, 34433 Istanbul, Turkey; ^4^School of Medicine, Department of Dermatology, Selçuk University, 42075 Konya, Turkey; ^5^School of Medicine, Department of Dermatology, Bulent Ecevit University, 67600 Zonguldak, Turkey

## Abstract

*Background*. The aim of this study was to compare the cytomorphometric characteristics of the buccal cells of Behçet's disease patients with those of healthy controls.* Methods*. This case-control study compared a group of 30 patients with Behçet's disease with an age- and gender-matched control group of 30 healthy individuals. The buccal mucosal smears were stained using the Papanicolaou technique for cytomorphometric analyses. The nuclear and cytoplasmic areas were evaluated using digital image analysis; the ratio of nuclear to cytoplasmic areas and nuclear roundness are presented.* Results*. The nuclear and cytoplasmic areas of the BD patients' cells were significantly smaller than those of the healthy controls' cells, while the nucleus-to-cytoplasm ratio and neutrophil infiltration rate did not differ significantly between the groups. However, the nuclear area, cytoplasmic area, nucleus-to-cytoplasm ratio, and nuclear roundness factor were significantly higher in patients without aphthae. The neutrophil infiltration rate did not differ significantly in patients with or without aphthae.* Conclusion*. Behçet's disease can produce cytomorphometric changes in buccal cells that are detectable by exfoliative cytology and cytomorphometric analysis techniques.

## 1. Introduction

Behçet's disease (BD) is a multisystemic inflammatory disorder characterized by oral and genital ulcers along with cutaneous, ocular, arthritic, vascular, central nervous system, and gastrointestinal involvement [1]. Its geographical distribution is widespread throughout the Mediterranean region and Asian countries. The disease is believed to have been historically transported via the ancient Silk Road [1]. The prevalence rate for BD in Turkey ranges from 8 to 42 in 10000 persons [2–4]. The etiology of BD is unknown, and hence genetic, infectious, and autoimmune components have all been presumed to be responsible for this disease [5]. As there are no pathognomonic clinical or laboratory findings for BD, it must be diagnosed upon clinical grounds according to the International Criteria for Behçet's Disease (ICBD) [1]. Early diagnosis of BD is important for controlling the complications of the disease through prompt institution of therapeutic interventions [1].

During the course of chronic diseases associated with inflammation, oral mucosal cytomorphometric changes occur [6–8]. BD often initially affects the oral mucosal surface, with oral aphthae as the initial manifestation in 70% of patients. The major clinical symptom of BD is also oral ulceration, which is present in 97–99% of patients [1]. Oral exfoliative cytology is a widely used technique for determining abnormal cells. This laboratory examination method is easy to perform, simple, and considered acceptable by patients [6]. This method is useful for evaluating the cellular alterations produced by systemic illnesses and infectious diseases [9–12].

We have not encountered any previous study in the literature evaluating the cytomorphometric characteristics of the buccal mucosal cells of BD patients. In the present study, we aimed to determine the relevance of quantitative exfoliative cytology in BD, describe the cytomorphometric characteristics of buccal mucosal cells in BD patients, and compare those characteristics with those of cells in healthy control subjects.

## 2. Material and Methods

This case-control study was performed on two groups. The study group consisted of 30 patients with BD whose diagnosis and follow-up were provided at the Dermatology outpatient clinic. The control group consisted of 30 age- and gender-matched healthy counterparts who received a periodical health examination at the Family Medicine outpatient clinic. The study was approved by the local ethics committee for human research and all of the participants have given their informed consent.

### 2.1. Patient Selection Criteria

A questionnaire was completed for each subject to collect information on past medical history, alcohol use, drug use, and smoking habits. Smokers, drug or alcohol addicts, and users of any other illicit drugs were excluded from the study and control groups. Patients and healthy control subjects were excluded from the study if they had atherosclerotic cardiovascular diseases, hyperlipidemia, recurrent aphthous stomatitis, anemia, or diabetes mellitus (according to history or complete physical examination and laboratory findings when necessary). None of the subjects had any malignancies or other chronic illnesses other than BD. The diagnosis of BD was established according to the ICBD criteria [1] (Table 1) at the Dermatology outpatient clinic. Patients scoring ≥ 4 according to the ICBD were diagnosed as having BD. The diagnostic procedure was carried out by three of the authors (Rafet Koca, Cevdet Altinyazar, and Serdar Bostan). The study samples were collected prior to the commencement of drug treatment for BD. Subjects of both groups had clinically healthy oral mucosa. The oral hygiene of the participants was evaluated by the presence of mucosal sensitivity, stomatitis, halitosis, xerostomia, gingival hemorrhage, and active carries. Patients and healthy controls having one of these signs were excluded from the study.

### 2.2. Sampling Process

Study organization and sampling from the study subjects were performed by one of the authors (Erol Aktunc), while the study design and technical efficiency of the slides were assessed by another author (Zehra Safi Oz). Salivary pH was measured with Whatman indicator paper (EU). The oral mucosa was dried with a gauze swab to remove surface debris and excess saliva. The sampling was carried out from the buccal mucosa using a cytobrush (Gynetics, Belgium). The freshly obtained specimens were streaked on glass slides and fixed in 95% ethyl alcohol. They were then stained using the Papanicolaou technique for cytomorphometric examination.

### 2.3. Cytomorphometric Examination

**Figure 1:**
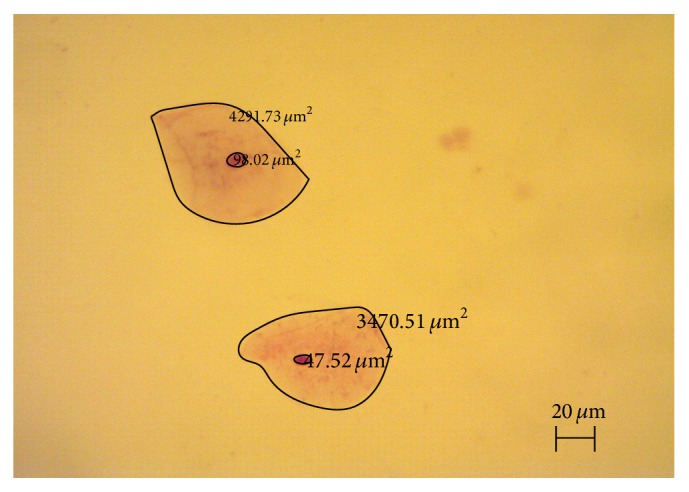
The encirclement of the nuclear and cytoplasmic boundaries of the cells on digitally obtained images (×100).

The study parameters examined included nuclear area (NA), cytoplasmic area (CA), ratio of nuclear area to cytoplasmic area (N/C), nuclear roundness factor (NRF), nuclear length (NL), nuclear width (NW), and nuclear perimeter (NP). The cytomorphometric examinations were carried out by two of the authors (Sibel Bektas, Zehra Safi Oz) blinded to the study and control groups. The cytological analysis was performed using digital photographs obtained from the slides using an Olympus BX 51 Microscope (Tokyo, Japan) and a mounted Leica DFC-280 digital camera (Germany). Measurements of the nuclear and cytoplasmic areas were calculated on digital images using a line encircling the nuclear and cytoplasmic boundaries of the cells (Figure 1).

### 2.4. Statistical Analysis

Statistical analysis was performed using SPSS for Windows (SPSS Inc., Version 22.0, Chicago, IL, USA). Continuous variables with normal distribution were presented as mean ± standard deviation and those that do not were presented as median (range). The normality of distribution for continuous variables was tested by the Shapiro-Wilk test. Mean values for the normally distributed continuous variables were compared using Student's *t*-test; otherwise, the Mann-Whitney *U* test was used. Categorical variables were presented as frequency and percent values, and the differences between groups were compared using the chi-squared test. *p* values < 0.05 were considered significant.

## 3. Results

The mean age of the study groups was comparable, 41.2 ± 10.4 years for BD patients and 37.9 ± 12.6 years for control subjects (*p* = 0.282). The female/male ratio also did not differ significantly between the BD patients (female/male = 7/23) and control subjects (female/male = 12/18) (*p* = 0.267). A group of younger and predominantly female BD patients (*n* = 18) had aphthous lesions on their oral mucosa during the study period. The median age of BD patients with aphthae was 40 (21–58), whereas the median age of BD patients without aphthae was 47 (31–60) (*p* = 0.034). The female/male ratio was 7/11 in BD patients with aphthae, whereas it was 0/12 in patients without aphthae (*p* = 0.024).

A total of 1500 cells from BD patients and 1500 cells from control subjects were digitally analyzed. The cytomorphometric measurements and the presence of polymorphonuclear leucocyte (PMNL) infiltration are depicted in Table 2. The NA and CA of the BD patients were found to be significantly smaller than those of the healthy controls (*p* < 0.05). The N/C ratio and PMNL infiltration rate did not differ significantly between the groups (Table 2). The NRF was higher in the BD group, while NL, NW, and NP were smaller in the BD group than those of the control group (all *p* < 0.001).

The cytomorphometric measurements of 900 cells from BD patients with aphthae and 600 cells from BD patients without aphthae were analyzed in Table 3. The NA, CA, N/C ratio, and NRF were significantly higher in patients without aphthae. The PMNL infiltration rate did not differ significantly between patients with or without aphthae.

## 4. Discussion

The main purpose of this study was to investigate the cytomorphometric characteristics of buccal epithelial cells in BD patients and to compare them with those of healthy control subjects using the Papanicolaou staining technique.

We observed the presence of significantly smaller buccal mucosal cells and concomitantly smaller nuclei in BD patients, without any significant PMNL infiltration. Cytoplasm and nuclei of buccal mucosal cells in BD patients with aphthae were also smaller than those of the patients without aphthae, again without any significant PMNL infiltration.

In the present study, nuclear irregularity significantly differed between BD patients and controls and between BD patients with aphthae and without aphthae. The degree of nuclear roundness has previously been suggested as a discriminating feature of cellular morphometric alterations in a number of diseases such as immune-mediated thrombocytopenic purpura, benign prostatic hyperplasia, prostatic carcinoma, and oral squamous cell carcinoma [10, 13–15].

The cytomorphometric measurements of the oral mucosal epithelial cells may be affected by patient characteristics such as age and gender, systemic diseases like anemia and diabetes, or local irritating agents such as tobacco, alcohol, illicit drugs, and infectious diseases [6–11, 16–19]. We presume that the quantitative cytomorphometric changes disclosed in the present study may be attributable primarily to BD itself as the confounding factors for buccal cellular morphometric alterations mentioned previously were not present in the subjects in either our study or control groups.

BD is known to be a multisystemic perivasculitis, having a course of inflammatory papulopustular cutaneous lesions [1]. It is known that, in early lesions of BD such as aphthae, pathergy reactions, and uveitis, significant neutrophil infiltration is evident [20]. More than half of the patients in our BD group had oral aphthae present during the sampling period, yet the PMNL infiltration rate was low and did not differ significantly from the control group. We presume that the presence of cytomorphometric alterations but the absence of significant PMNL infiltration in the present study may be related to the changes in oxidative burden of the tissues caused by the disease itself. In several studies, oxidative stress biomarkers were found to be higher in BD patients than in a normal population, and they were suggested to be disease markers for BD [21–23]. A cross-reaction of antibodies to* Streptococcus sanguinis* with bodily proteins may also be responsible for aphthae formation in BD patients. Since all of the BD patients without aphthae during the study were male, the importance of hormonal differences should also be considered in aphthae formation in female patients.

To the best of our knowledge, the present study is the only one to investigate the buccal mucosal cellular cytomorphometric alterations in BD patients and compare them with those of healthy individuals. Therefore, we are unable to compare our findings directly with the results of any other study.

In summary, this study analyzed the quantitative cytomorphometric characteristics of the buccal mucosal cells in BD patients and compared them with those of healthy control subjects. We concluded that BD itself likely produces cytomorphometric alterations in the buccal mucosal squamous epithelium. These alterations are detectable by cytomorphometric analysis through exfoliative cytology. The cytomorphometric view of the mucosal cells in BD patients presented in this study will contribute to the understanding of the effects of BD on the oral mucosa.

## Figures and Tables

**Table 1:** International Criteria for Behçet's Disease [1].

	Clinical manifestation	Point
1	Oral aphthosis	2
2	Genital aphthosis	2
3	Skin manifestations	1
4	Ocular manifestations	2
5	Vascular manifestations	1
6	Pathergy phenomenon	1

**Table 2:** Comparison of the cytomorphometric parameters and the presence of polymorphonuclear leukocytes (PMNL) in Behçet's disease and control groups.

Cytomorphometric parameters Median (range)	Behçet's disease group (*n* = 30)	Control group (*n* = 30)	*p* value
Nuclear area (*µ*m^2^)	143.5 (13.9–697.4)	369.2 (53.4–998.5)	<0.001^†^
Cytoplasmic area (*µ*m^2^)	5734.1 (1461.4–9968.7)	13688.5 (4093.8–44469.3)	<0.001^†^
Nucleus/cytoplasm ratio	0.03 (0.005–0.11)	0.03 (0.004–0.12)	0.475^†^
NRF^*∗*^	0.94 (0.70–0.98)	0.89 (0.38–0.98)	<0.001^†^
NL (*µ*m)^*∗∗*^	16.1 (4.5–35.5)	26.8 (9.9–46.2)	<0.001^†^
NW (*µ*m)^*∗∗∗*^	12.0 (4.3–26.2)	19.2 (6.3–35.8)	<0.001^†^
NP (*µ*m)^*∗∗∗∗*^	44.3 (13.5–83.3)	72.3 (26.8–122.1)	<0.001^†^
PMNL			
Present	3	9	0.53^‡^
Absent	27	21	

^*∗*^Nuclear roundness factor, ^*∗∗*^nuclear length, ^*∗∗∗*^nuclear width, ^*∗∗∗∗*^nuclear perimeter, ^‡^Pearson chi-squared test, and ^†^Mann-Whitney *U* test.

**Table 3:** Comparison of the cytomorphometric parameters and the presence of polymorphonuclear leukocytes (PMNL) in Behçet's disease patients with and without aphthae.

Cytomorphometric parameters Median (range)	Behçet's disease patients having aphthae (*n* = 18)	Behçet's disease patients not having aphthae (*n* = 12)	*p* value
Nuclear area (*µ*m^2^)	131.1 (13.9–730.2)	188.2 (38.4–1026.7)	<0.001^†^
Cytoplasmic area (*µ*m^2^)	5709.5 (717.6–27260.4)	7067.5 (2316.9–22819.9)	<0.001^†^
Nucleus/cytoplasm ratio	0.02 (0.01–0.06)	0.03 (0.01–0.07)	<0.001^†^
NRF^*∗*^	0.9 (0.7–0.9)	0.9 (0.8–0.9)	<0.001^†^
NL (*µ*m)^*∗∗*^	15.7 (4.5–143.5)	18.1 (8.8–132.8)	<0.001^†^
NW (*µ*m)^*∗∗∗*^	11.4 (4.3–109.1)	14.2 (5.4–116.8)	<0.001^†^
NP (*µ*m)^*∗∗∗∗*^	42.7 (13.5–389.4)	50.7 (23.5–378.3)	<0.001^†^
PMNL infiltration			
Present	2	1	0.548^‡^
Absent	16	11	

^*∗*^Nuclear roundness factor, ^*∗∗*^nuclear length, ^*∗∗∗*^nuclear width, ^*∗∗∗∗*^nuclear perimeter, ^‡^Pearson chi-squared test, and ^†^Mann-Whitney *U* test.
